# Supplementary material for: Pumilio protects Xbp1 mRNA from regulated Ire1-dependent decay
Source: Nat Commun. 2022 Mar 24;13:1587. doi: 10.1038/s41467-022-29105-x (PMC8948244; doi:10.1038/s41467-022-29105-x)
Supplement: Supplementary file 1 — Supplementary Information [file 41467_2022_29105_MOESM1_ESM.pdf]

Pumilio protects Xbp1 mRNA from regulated Ire1-dependent decay

Fátima Cairrão[1], Cristiana C Santos[1], Adrien Le Thomas[2], Scot Marsters[2], Avi Ashkenazi[2] and Pedro M. Domingos[1]

1 - Instituto de Tecnologia Química e Biológica, Universidade Nova de Lisboa, Av. da República, 2780-157 Oeiras, Portugal 2 - Cancer Immunology, Genentech, Inc., 1 DNA Way, South San Francisco, CA 94080, USA

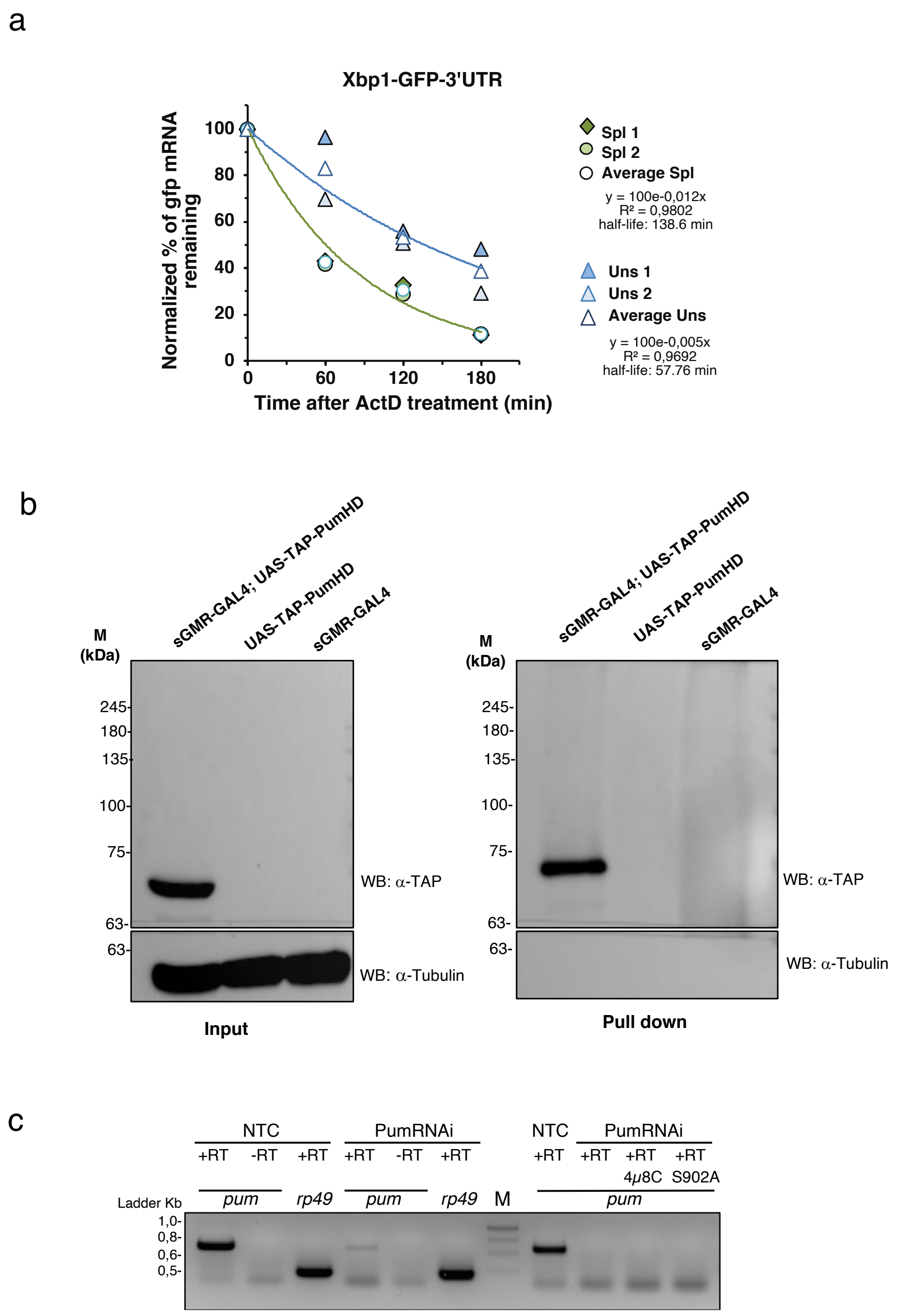

Supplementary Figure 1 - The 3'UTRs of Xbp1^{unspliced} and Xbp1^{spliced} differ in their stability effect

a) The stability of the GFP reporters fused with the 3'UTRs of Xbp1^{spliced} or Xbp1^{unspliced} was assessed by RT-PCR, using primers specific for *gfp* and *rp49* mRNAs (control). The levels of mRNA reporter were normalized to those of *rp49* mRNA, and averages and standard deviations from three independent experiments are plotted. The 3'UTR of Xbp1^{unspliced} shows a higher stability effect of the reporter half-life (2 fold stabilization) relative to the spliced form. Data are presented as mean ± SD. Spliced (green, n=2); Unspliced (blue, n=2). n = 2 biological independent experiments.

b) Immunoblot from adult *Drosophila* eyes expressing TAP-PumHD (sGMR-GAL4; UAS-TAP-PumHD) and the 2 parental control lines. Both input and pull-down samples were run and probed with anti-TAP and anti-Tubulin (loading control) antibodies. n = 2 independent experiments.

c) RT-PCR results from S2 cells treated with PumRNAi or LacZ RNAi (NTC - non-target control) to assay for the efficiency and specificity of PumRNAi treatments, using primers specific for *pumilio (pum)* or the loading control *rp49*. RNA samples were treated (+RT) or not (-RT) with reverse transcriptase.
M – molecular weight markers. n ≥ 3 of independent experiments.

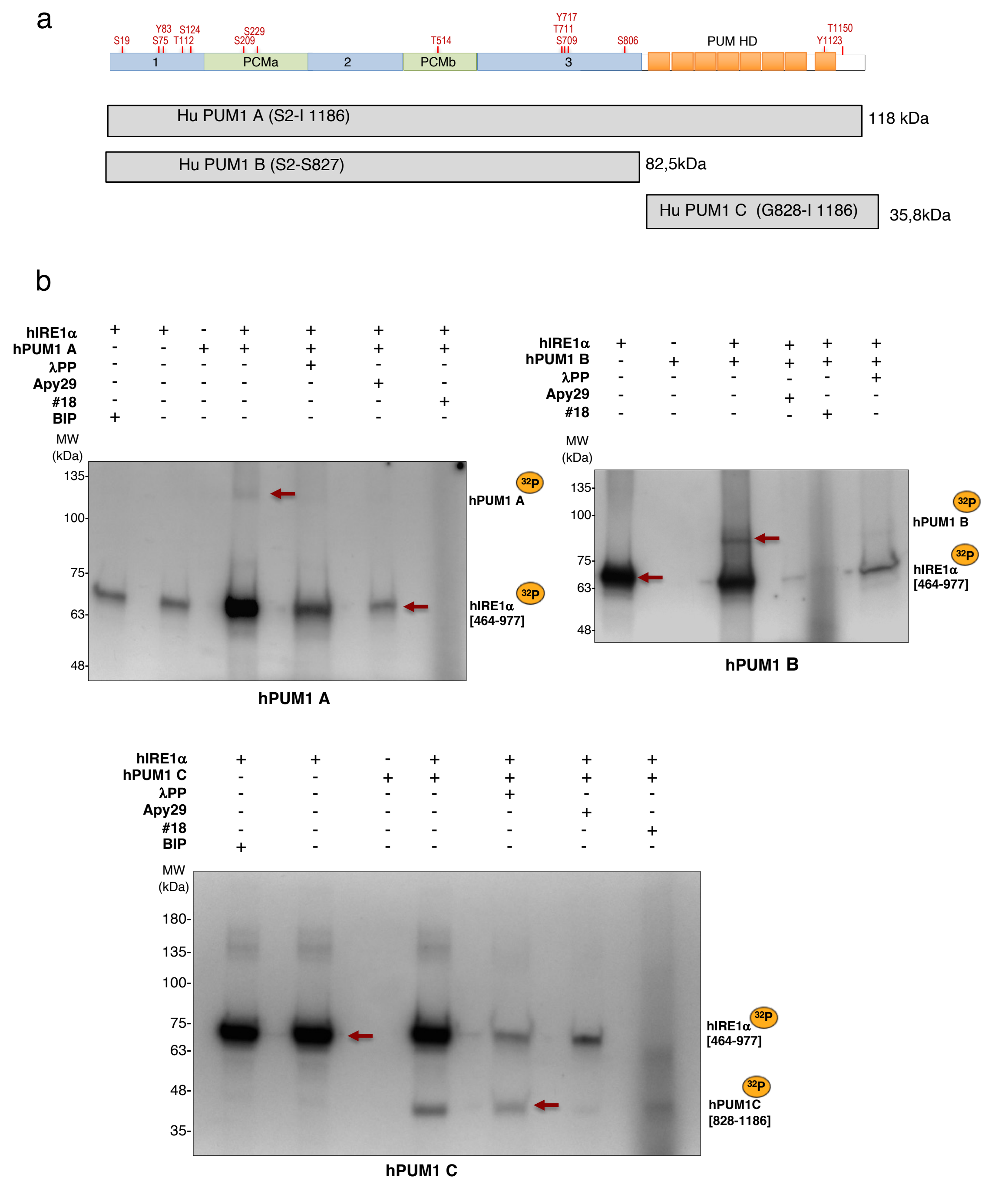

Supplementary Figure 2 - hIRE1α KR domain phosphorylates hPUM1 in vitro

a) Schematic representation of domains and putative phosphorylation sites present in Human PUM1. **b**) *in vitro* radioactive kinase assay with γ-[^{32}P]ATP of purified hPUM1 forms incubated with hIRE1α KR domain. The specificity of phosphorylation was monitored by treatment with λPP or incubation with specific inhibitors of IRE1 kinase activity (Apy29 and compound#18). Arrows express hPUM1 phosphorylated forms corresponding to the expected MW of each purified protein. Treatment with kinase inhibitors decreased the amount of detected radioactive protein bands. n≥2 of independent experiments. Source data file is provided.

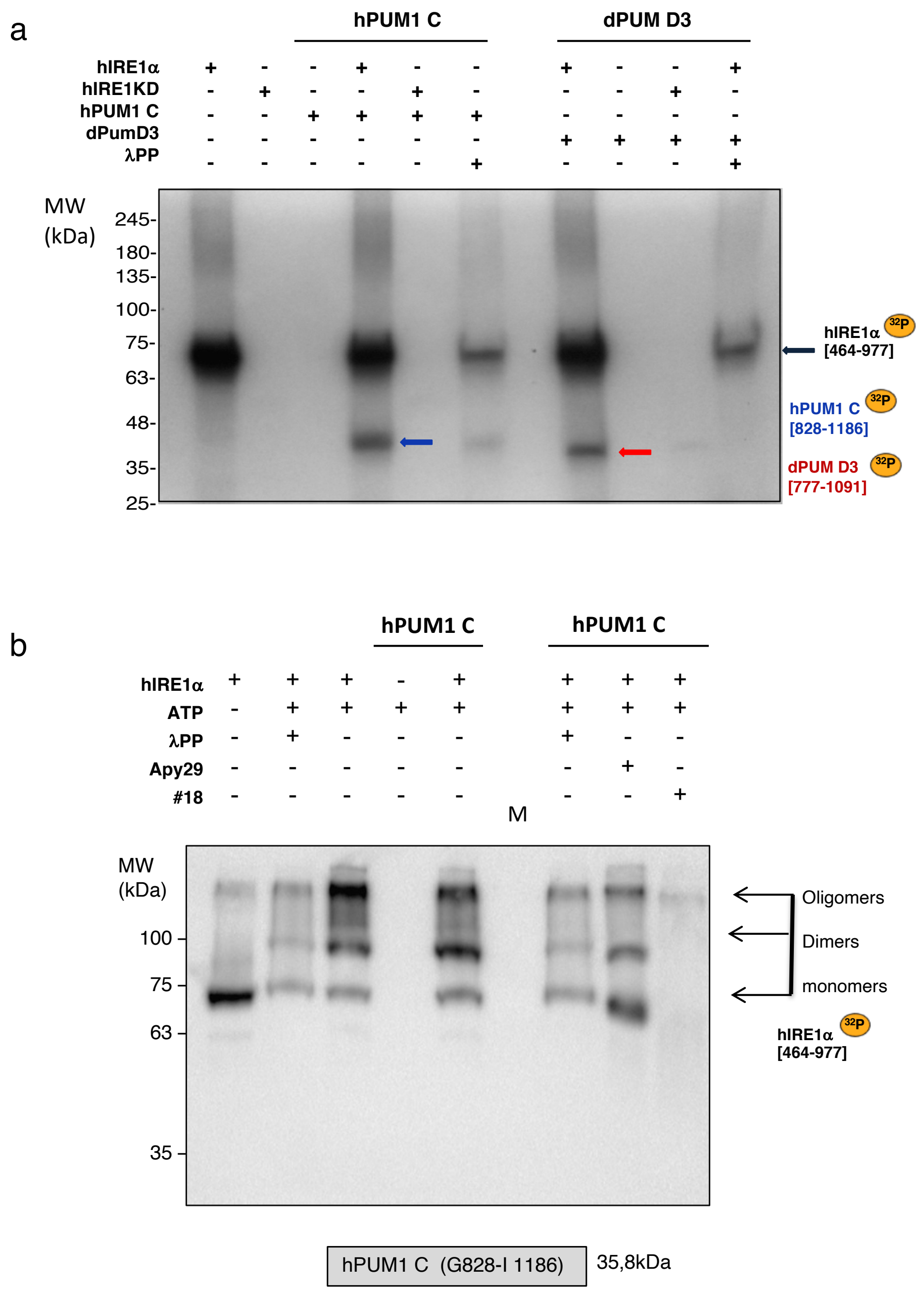

Supplementary Figure 3 - Validation of *in vitro* phosphorylation hPUM1-C and dPUM-D3
a) *In vitro* phosphorylation hPUM1-C domain and dPUM-D3 domain by hIRE1α KR using the control kinase-dead Ire1 (hIRE1KD, D688N). Assays were conducted as described before. Arrows denote phosphorylated forms of hPUM-C (Blue arrow) and dPUM-D3 purified proteins (red arrow). Auto-phosphorylation of hIRE1α KR is denoted by a black arrow. n=3 of independent experiments**.**
b) Phostag immunoblot of phosphorylation of hPUM-C with an antibody specific for phosphorylated IRE1(anti-rabbit Phospho-IRE1). The specificity of phosphorylation was monitored by treatment with λPP or incubation with specific inhibitors of IRE1α kinase activity (Apy29 and compound #18) and a reaction lacking ATP (first lane). The phosphorylation state of hIRE1α KR is decreased in control reactions as expected compared to the reactions containing hIRE1α+ATP or hIRE1α+hPUM-C+ATP. An additional control was made by conducting a kinase assay of hPUM-C without incubation with the hIRE1α KR domain. M – Protein molecular weight marker lane. n ≥ 2 of independent experiments. Source data file is provided.

a

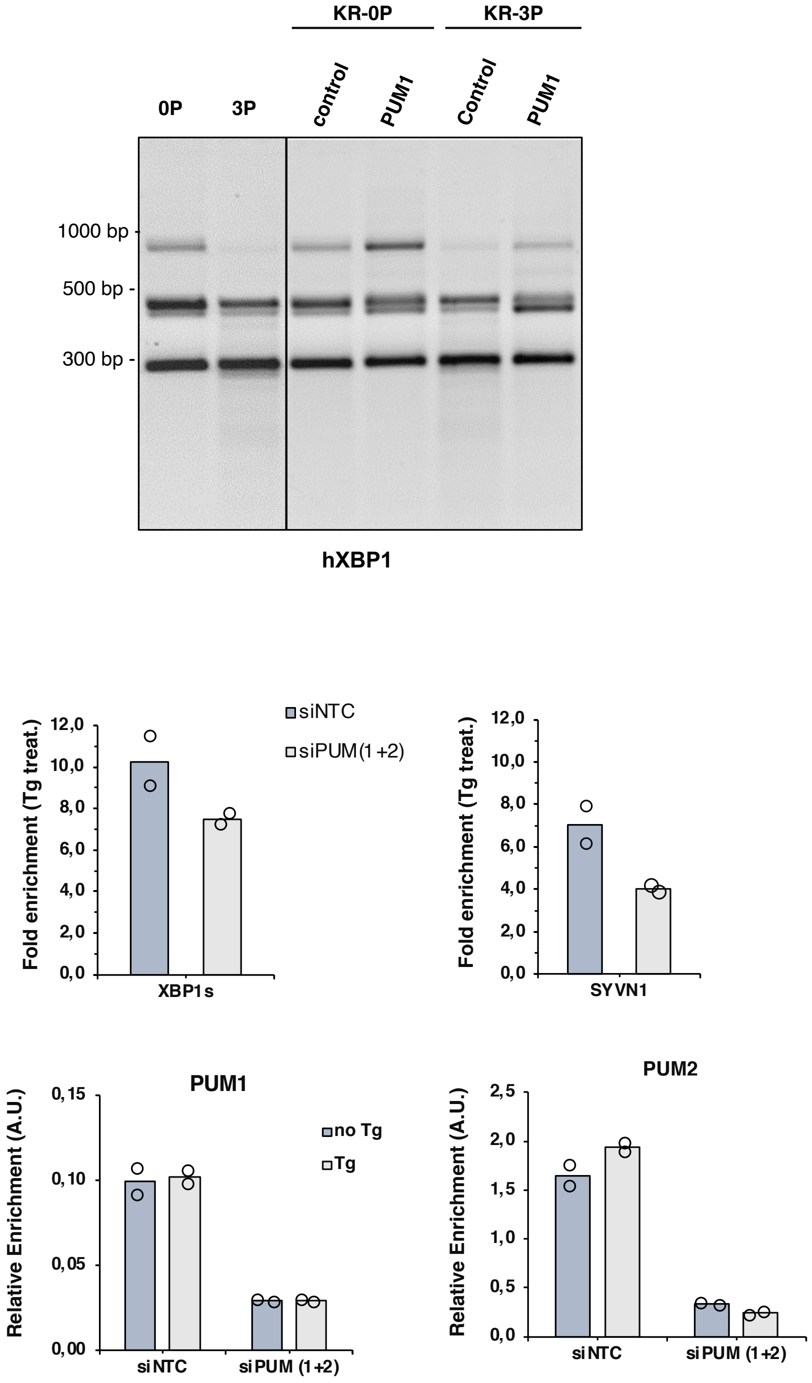

Supplementary Figure 4 – PUM1 protects human XBP1 from regulated Ire1-dependent decay

a) *In vitro* transcripts of human XBP1 were incubated with non-phosphorylated (0P) and phosphorylated (3P) forms of purified hIRE1α KR. Human PUM1 FL protects XBP1 RNA from Ire1 dependent non-canonical decay, but does not impair IRE1 dependent XBP1 splicing. n = 3 of independent experiments.

b) RT-qPCRs for human XBP1s and the XBP1s target SYVN1 from human MDA-MB-231 cells treated with Thapsigargin (Tg) or vehicle control (DMSO) after transfection with a non-targeting control (siNTC) siRNA, or siRNAs for PUM1 and PUM2 (siPUM1+2). Knock-down efficiency of siPUM1+2 was evaluated by RT-qPCR for PUM1 and PUM2. n= 2 biological independent experiments. Source data file is provided.

Supplementary Table 1

Primers used for plasmids and mutants used in this study

**Pum D1-V5-H6** in pIZ	FW dom.1 + pcma PUM	CGAATTTAAAGCTTCAAAATGAAGTTTTTGGGTG
	Rev. dom.1 + pcma PUM	GCCCTCTAGACTCTCTGCAGGTGATGGTTG
**PumD3-V5-H6** in plz	Fw pcmb + dom. 3 PUM	CGAATTTAAAGCTTCAAAATGTATGGCGTAGCACCATGG
	Rev. Fw pcmb + dom. 3 PUM	GCCCTCTAGACTTCCTGGCTGTGGGGCAC
**Pum D1D3-V5-H6** in plz	FW dom.1 + pcma PUM	CGAATTTAAAGCTTCAAAATGAAGTTTTTGGGTG
	Rev. Fw pcmb + dom. 3 PUM	GCCCTCTAGACTTCCTGGCTGTGGGGCAC
**PumilioV5-H6** in pET26b	Nde1-pumilio Fw	GGATTCCATATGAAGTTTTTGGGTGGTAACGATG
	EcoR1-V5His6Pum	GGAATTCCAGTCAGATAAACTCAATGGTG
**PumD3-V5-H6** in pET26b	Nde1-D3	GGATTCCATATGTATGGCGTAGCACC
	EcoR1-V5His6Pum	GGAATTCCAGTCAGATAAACTCAATGGTG
**PumHD-V5-H6** in pET26b	Nde1-PumHD FW	GGATTCCATATGAGATCTCGCCTTCTCGAAG
	EcoR1-V5His6Pum	GGAATTCCAGTCAGATAAACTCAATGGTG
**Pum D1D3-V5-H6** pET26b	Nde1-pumilio Fw	GGATTCCATATGAAGTTTTTGGGTGGTAACGATG
	EcoR1-V5His6Pum	GCCCTCTAGACTTCCTGGCTGTGGGGCAC
**Xbp1-3'UTR**	Xbp1-BamHI	CGGGATCCTTTATATTTTGAATATTTTTAG
	Xbp1-3UTR un FW	cggtaccATGTTGAGCAGGAACAGG
	Xbp1-3UTR sp FW	cggtaccGTTTTTTAGTTGATAGGCTG
	Xbp1-revGH	gctctagaGcGTGACACTATAGAACTCGAG
**PumPRE Mutants** in pRM-GFP	PumS3-R	TAATCATAAGCCTATGTGGTTAGAG
	PumS3-Fmut	CTCTTATAacaAacaATTGTGCTCAAAG
	Pum s1 Fw	GCTTTATATATGATACTGTAGTTAGAGCG
	Pum s1rev mut	CTTCGATTATGAATTTATGTTGTGCCTATC
**new reporter Xbp1-HA-GFP**	RV xbp1 HA	ggc tcg cat aat ctg gaa cat cgt acg ga t aat aaG ATA TCT GCG AGC AGA CTT TCG GC
	FW xbp1 HA	tat ccg tac gat gtt cca gat tat gcg ag c ctc tGA CCC CGG TCG CCA CCA TGG TGA GC
	eGfp-stop -fw	GGACTCAGATCTCGAGGGTACCGATCTG
	eGFP-stop-rev	GGTACCCTCGAGATCTGAGTCCTTACTTGT
	eGFP-ecoR5 Fw	GGGATATCGTGAGCAAGGGCGAGGAGCTG
	eGfp-stop -fw	GGACTCAGATCTCGAGGGTACCGATCTG
	eGFP-stop-rev	GGTACCCTCGAGATCTGAGTCCTTACTTGT
	eGFP-ecoR5 Fw	GGGATATCGTGAGCAAGGGCGAGGAGCTG
**Pumilio phosphomutants**		
	FwmutT537A	GGC TTC AAT CGC GCA CCT GGT GCA CGT CAA CCA GCA CCT GCA GAG
**T**537A; **S**540A;**S**544A	RevmutT537A-S540A-S544A	CTC TGC AGG TGC TGG TTG ACG TGC ACC AGG TGC GCG ATT GAA GCC
S902A	SITmutD3 B FW	CGTTGACGGGTCGCCGCGACGCCTTCGACCGCAGCACC
	SITmutD3 B rev	GGTGCTGCGGTCGAAGGCGTCGCGGCGACCCGTCAACG
	Mut Dom1 nFW	GCC GAT TCG ACT TGC GCC AAA GTG GTT G
	MutDom1 nREV	GGG GGC CAG TAT GCC CGC ATT GCC GCT
	MutDom3A nFW	CCC GCC CAG CAG GGT GCC GAG AAT C
	MutDom3A nREV	GGC GAG GGG GCG GCG CGG CTG CGT T

qPCR primers used

	qPCR primers used	Target
GFP-FW1	GGCTACGTCCAGGAGCGCACCATCTT	gfp
GFP-Fw2	AGACCCCAACGAGAAGCG	gfp
Xbp1 intron	GGGTCAGCTTTGGATGCTGCAGA	Unspliced
Xbp1 FUns	CCAGACATCGCGTGACCGCAAG	Unspliced /spliced
Xbp1 RUnsSp	CACAACTTTCCAGAGTGAGGCCAG	Unspliced /Spliced
RP49-Fc1	AGATCGTGAAGAAGCGCACCAAGC	RP49
RP49-Fc2	GCACCAGGAACTTCTTGAATCCGG	RP49
Rev2 xbp1 spl/Uns	GCGCTTGACGTCGAACTCTTCGT	Spliced
Intron fw xbp1	CTGCAGCATCCAAAGCTGACCC	Unspliced
xbp1 spliced fw	CCAACCTTGGACCGCAGGG	spliced
xbp1s_Fw2	CCAACCTTGGATCTGCCGCA	spliced
xbp1S_Fw3	CAACCTTGGATCTGCCGCAGGGTA	spliced
rp49_Fw3	CCCAAGATCGTGAAGAAGCGCACCAAGC	rp49
rp49_rev4	GCACGTTGTGCACCAGGAACTTCTTGAATCC	rp49
Hsc3_forw	TGTCACCGATCTGGTTCTTCAGGC	Hsc3
Hsc3_rev	GTCCCATGACCAAGGACAACCATC	Hsc3
Acat2-fw-qPCR	CATCACTGCCGAGAATCTG	Acat2
Acat2-rv-qPCR	CTGAGCTTCTGTATACCCTCG	Acat2

EMSA assays

RNA oligos

Name	Sequence	Bases	End Modification 3'
pum1site-wt-3BioTEG	GAUAGGCUGUACAUAAAUUCAUAAUCG	27	Biotin-TEG
pum1site-wt	GAUAGGCUGUACAUAAAUUCAUAAUCG	27	
pum1site-mut	GAUAGGCACAACAUAAAUUCAUAAUCG	27	

Reagents/Kits

Designation	Source or reference	Identifiers	Additional information
Amersham™ Hybon N+	GE Healthcare	RPN2222B	
ECL Streptavidin-HRP Amersham™	GE Healthcare	RPN 1231	(1:1000)
1xTBS 1% Casein blocker	BIO-RAD	#1610782	

Primers used for dsRNAi experiments

T7 primer	TAATACGACTCACTATAGGG
T7-rnai Pum Fw	cgTAATACGACTCACTATAGGGgaaacttccttctcga atcgc
T7-rnai Pum Rv	cgTAATACGACTCACTATAGGGatcgtaggctaattca tgcagc
PUM Fw	gaaacttccttctcgaatcgc
PUM Rev	atcgtaggctaattcatgcagc
T7-Rnai LacZ Fw	GGATCC**TAATACGACTCACT ATAGGG**TGACGTCTCGTTGCTGCATAAAC
T7-RNAi LacZ ReV	GGATCC**TAATACGACTCACTATAGGG**GGCGTTAAAGTTGTTCT GCTTCATC

Supplementary Table 2

Designation	Source or reference	Identifiers	Additional information
**Antibodies**			
V5 Tag Antibody (mouse monoclonal)	Invitrogen	(R960-25); RRID: AB_2556564	1:1000-1:5000
TAP-tag Antibody (Rabbit-pAb)	GenScript	#A00683	1:2000
ELAV (Anti-Rat)	Developmental Studies Hybridoma Bank (DSHB),	DSHB Cat# 1ea AB_528217	(1:400)
anti-alpha-tubulin (mouse monoclonal)	Developmental Studies Hybridoma Bank (DSHB),	DSHB Cat# 12G10 RRID:AB_1157911	(1:50)
Anti-HA (mouse monoclonal)	Biolegend Clone [16B12]	Covance Catalog# MMS-101R	(1:2000)
Anti-HA (rat monoclonal)	Chromotek Clone [7C9]	HA antibody [7C9] RRID: AB_2631399	(1:1000)
Anti-GFP (rat monoclonal)	Chromotek Clone [3H9]	3h9-100 RRID: AB_10773374	(1:1000)
Ire1 Phospho	Genentech	NA	(1: 1000)
Anti-mouse IgG HRP<br>Anti-Rabbit IgG HRP<br>Anti-Rat IgG HRP	GE Healthcare<br>Sigma<br>Sigma	NXA931<br>A8275<br>A9037	(1:5000)<br>(1:10000)<br>(1:2000)
Cy3-conjugated donkey anti-rabbit IgG (H+L)	Jackson ImmunoResearch laboratories	#711-165-152	(1:400)
Cy3 AffiniPure Donkey Anti-Rat IgG (H+L)	Jackson ImmunoResearch laboratories	#712-165-150	(1:400)
Cy3-conjugated donkey anti-mouse	Jackson ImmunoResearch laboratories	#715-165-150	(1:400)
Alexa Fluor® 488 AffiniPure Donkey Anti-Mouse IgG (H+L)	Jackson ImmunoResearch laboratories	715-545-151	(1:400)
Goat anti-Rabbit IgG Dye 650	Advansta	R-05761-250	(1:400)
Alexa Fluor® 647 AffiniPure Donkey Anti-Rat IgG (H+L)	Jackson ImmunoResearch laboratories	712-605-153	(1:400)
Alexa Fluor® 647 AffiniPure Donkey Anti-Mouse IgG (H+L)	Jackson ImmunoResearch laboratories	715-605-151	(1:400)
Goat anti-Mouse IgG Dye 650	Advansta	R-05764-250	(1:400)
**Reagents/Kits**			
NucleoBond ® Xtra Midi kit for transfection-grade plasmid DNA	MACHEREY-NAGEL	MN740410	
NZYMiniprep	nzytech	MB01002	
NZY Total RNA isolation kit	nzytech	MB13402	
TRIzol™Reagent	Invitrogen	#15596026	
RiboMAX™ Large Scale RNA Production System	Promega	# P1300	
RNA Clean & Concentrator ™- 5	Zymo Research	R1015	
RNasin® Ribonuclease Inhibitor	Promega	N2511	
Amersham™ Hybond PVDF Blotting Membrane	GE Healthcare	# 10600021	
Amersham™ ECL Chemiluminescent HRP Substrate	GE Healthcare	RPN2232	
ATP, [γ-32P]- 3000Ci/mmol, 250μCi , 5mCi/mL	PerkinElmer	PELSBLU502H250UC	
Amersham Hyperfilm™ ECL	GE Healthcare	#28906836	
Microseal B Adhesive Seals	BIO-RAD	MSB-1001	
iCycler iQ® PCR Plates 96-well	BIO-RAD	# 2239441	
Effectene Transfection Reagent	QIAGEN	# 1054250	
Penicillin/streptomycin	ThermoFisher Scientific	Cat #15140122	
FBS Hyclone	Biowest	SV30160.03	
Schneider's medium	Biowest	L0207-500	
NZYColour Protein Marker II	nzytech	MB09002	
cOmplete™, Mini, EDTA-free Protease Inhibitor Cocktail	Roche	4693159001	
Phos-tag™ Acrylamide NARD institute	Wako-Chem Europe GmbH	AAL-107	
Halt Protease & Phosphatase Inhitor Cocktail, EDTA free	Thermo Scientific	#78441	
40% Acryllamide/Bis Solution 29:1	BIO-RAD	#1610146	
4–20% Mini-PROTEAN® TGX™ Precast Protein Gel	BIO-RAD	#456-1085	
7% Mini-PROTEAN® TGX™ Precast Protein Gel	BIO-RAD	#456-1025	
Slide-A-Lyzer MINI Dialysis devices	Thermo Scientific	#88404	
Amicon Ultra Centrifugal filter Devices	Merck	10K, 30K, 50K	
Rabbit IgG agarose beads	Sigma	A2909	
Ni-NTA Resin	G biosciences	#186-939	
V5 tagged Protein Purification Kit	MBL	code: 3317	
NZYTaq DNA polymerase	nzytech	MB00101	
Pfu DNA Polymerase	Fermentas	#EP0501	
Phusion High-Fidelity DNA Polymerase	ThermoFisher Scientific	F-530S	
NZYMutagenesis kit	nzytech	MB01201	
GreenSafe Premium	nzytech	MB13201	
GeneRuler 1kB DNA Ladder	ThermoScientific	#SM0311	
T4 Polynucleotide Kinase	NEB	M0201S	
T4 DNA Ligase	NEB	M0202S	
lambda phosphatase	NEB	P0753S	
Amgen Compound #18	Genentech		
Subitinib	Sigma-Aldrich	PZ0012	
Apy29	MedChemTronica	HY-17537	
4μ8c	Sigma-Aldrich	SML0949	
Actinomycin D	Sigma	A1410	
Vectashield Mounting medium with DAPI	Vector Laboratories, RRID:SCR_000821	VectorLabs: H-1200	
**Software**			
FIJI http://fiji.sc/	http://fiji.sc/	RRID:SCR_002285)	
GraphPad Prism	https://www.graphpad.com/	free trial	
https://astatsa.com/OneWay_Anova_with_TukeyHSD/	astatsa.com	2016 Navendu Vasavada	
Image Lab Software for Mac Version 6.1	Bio-RAD	SOFT-LIT-170-9690-ILSMAC-V-6-1	
CFX Manager™ Software	Bio-RAD	1845001	
Microsoft Excel version16.55	©2021Microsoft		

Supplementary Table 3 - MIQE checklist

ITEM TO CHECK	IMPORTANCE	CHECKLIST
**EXPERIMENTAL DESIGN**		
Definition of experimental and control groups	E	Non-targeting control (NTC), dsRNA against Pumilio versus control,
Number within each group	E	3-6 biological replicates and 3 technical replicates
Assay carried out by core lab or investigator's lab?	D	Investigator's lab
Acknowledgement of authors' contributions	D	
**SAMPLE**		
Description	E	Dmel-2 cells (S2 derivative):control and samples treated with dsRNAs
Volume/mass of sample processed	D	0,5 ml Dmel-2 cells (0.5-1 x10^6 cells) used in RNA isolation
Microdissection or macrodissection	E	n/a (cell culture)
Processing procedure	E	Investigator's lab
If frozen - how and how quickly?	E	cells processed immediately for RNA isolation,using RNA kit
If fixed - with what, how quickly?	E	n/a
Sample storage conditions and duration (especially for FFPE samples)	E	na
**NUCLEIC ACID EXTRACTION**		
Procedure and/or instrumentation	E	kit RNA extraction
Name of kit and details of any modifications	E	NZY Total RNA isolation kit (nzytech) / Quick-RNA Miniprep (Zymo Research)
Source of additional reagents used	D	
Details of DNase or RNAse treatment	E	1µl Turbo DNA (Ambion) per sample
Contamination assessment (DNA or RNA)	E	qPCR on RNA after turbo Dnase treatment control in qPCR (no RT)
Nucleic acid quantification	E	Nanodrop One
Instrument and method	E	Nanodrop One
Purity (A260/A280)	D	1.8 to 2.0
Yield	D	1-2µg total RNA
RNA integrity method/instrument	E	RNA quality evaluated with electrophoresis
RIN/RQI or Cq of 3' and 5' transcripts	E	N/A (used gel electrophoresis)
Electrophoresis traces	D	
Inhibition testing (Cq dilutions, spike or other)	E	Dilutions (standard curve efficiencies)
**REVERSE TRANSCRIPTION**		
Complete reaction conditions	E	RevertAid H Minus First Strand cDNA Synthesis Kit (Thermo/Fermentas)
Amount of RNA and reaction volume	E	0,5 to 1 µg RNA for RT, and 500 ng to 1 µg noRT 20 µl reaction volume
Priming oligonucleotide (if using GSP) and concentration	E	Random hexamers from 500 ng/µl stock - 50 ng/µl in annealing, 25 ng/µl final in 20 µl
Reverse transcriptase and concentration	E	2 U/µl
Temperature and time	E	42°C for 45 minutes
Manufacturer of reagents and catalogue numbers	D	Thermo Scientific (K1632)
Cqs with and without RT	D*	25ng of RNA sample after turboDNase treatment, C
Storage conditions of cDNA	D	stored at -20°C
**qPCR TARGET INFORMATION**		
If multiplex, efficiency and LOD of each assay.	E	n/a
Sequence accession number	E	Provided in qPCR primer information table
Location of amplicon	D	
Amplicon length	E	Provided in qPCR primer information table
In silico specificity screen (BLAST, etc)	E	NCBI Primer-BLAST ; Flyrnai.org
Pseudogenes, retropseudogenes or other homologs?	D	
Sequence alignment	D	
Secondary structure analysis of amplicon	D	
Location of each primer by exon or intron (if applicable)	E	Provided in qPCR primer information table
What splice variants are targeted?	E	Provided in qPCR primer information table
**qPCR OLIGONUCLEOTIDES**		
Primer sequences	E	Provided in qPCR primer information table
RTPrimerDB Identification Number	D	
Probe sequences	D**	
Location and identity of any modifications	E	n/a
Manufacturer of oligonucleotides	D	Stabvida https://www.stabvida.com
Purification method	D	Standard Desalting
**qPCR PROTOCOL**		
Complete reaction conditions	E	Standard reaction conditions recommended by SyberGreen (BioRad)
Reaction volume and amount of cDNA/DNA	E	10 µl reaction volume, 1/40 of cDNA from the 20µl cDNA reaction
Primer, (probe), Mg++ and dNTP concentrations	E	0.1 µM each primer, MgCl2 and dNTP concentration proprietary (Biorad)
Polymerase identity and concentration	E	SsoAdvanced Universal SYBR Green Supermix (2x concentrated, and contain dNTPs, $MgCl_2$, and DNA polymerase)
Buffer/kit identity and manufacturer	E	Biorad Cat Nª :1725271
Exact chemical constitution of the buffer	D	Exact buffer composition proprietary (Biorad)
Additives (SYBR Green I, DMSO, etc.)	E	SYBR Green
Manufacturer of plates/tubes and catalog number	D	BioRad, catalogue #HSP9601
Complete thermocycling parameters	E	Reported in Methods section
Reaction setup (manual/robotic)	D	Manual
Manufacturer of qPCR instrument	E	BioRad
**qPCR VALIDATION**		
Evidence of optimisation (from gradients)	D	Standard curves initially performed with Ta 65°C, then gradient or alternative Ta performed if efficiency not betw
Specificity (gel, sequence, melt, or digest)	E	Melt curves; amplicons were also evaluated using gel electrophoresis
For SYBR Green I, Cq of the NTC	E	Below detection limit (N/A for all primers)
Standard curves with slope and y-intercept	E	Performed standard curves for all primer sets; slope and y-intercept provided in qPCR primer information table
PCR efficiency calculated from slope	E	Efficiencies were between 84.7% and 103.9% and are provided in qPCR primer information table
Confidence interval for PCR efficiency or standard error	D	
r2 of standard curve	E	Provided in qPCR primer information table
Linear dynamic range	E	All measurements made within the linear range of detection determined from standard curves
Cq variation at lower limit	E	All assays were performed within the linear range of the standard curve for each primer set
Confidence intervals throughout range	D	
Evidence for limit of detection	E	All assays were performed within the linear range of the standard curve for each primer set
If multiplex, efficiency and LOD of each assay.	E	N/A
**DATA ANALYSIS**		
qPCR analysis program (source, version)	E	BioRad CFX Manager v 3.1; Fold change calculations done manually in Microsoft Excel
Cq method determination	E	Pffafl; not all primer sets within 5% efficiency of reference primer set
Outlier identification and disposition	E	N/A
Results of NTCs	E	Below detection limit: Ct was "N/A" for all primer sets
Justification of number and choice of reference genes	E	1 (Rp49) - constituitive ribosomal subunit within desirable Ct range (15-25)
Description of normalisation method	E	ddCT method
Number and concordance of biological replicates	D	3-4 biological replicates
Number and stage (RT or qPCR) of technical replicates	E	3 technical replicates, qPCR
Repeatability (intra-assay variation)	E	Triplicate measurements (technical reps), 3 independent trials
Reproducibility (inter-assay variation, %CV)	D	
Power analysis	D	
Statistical methods for result significance	E	Standard t-test (2-tailed, equal variance), reported in Source Data for each relevant figure
Software (source, version)	E	BioRad CFX Manager v 3.1
Cq or raw data submission using RDML	D	Cq values for individual samples can be found in Source data for each relevant figure

Table 1. MIQE checklist for authors, reviewers and editors. All essential information (E) must be submitted with the manuscript. Desirable information (D) should be submitted if available. If using primers obtained from RTPrimerDB, information on qPCR target, oligonucleotides, protocols and validation is available from that source.

*: Assessing the absence of DNA using a no RT assay is essential when first extracting RNA. Once the sample has been validated as RDNA-free, inclusion of a no-RT control is desirable, but no longer essential.

**: Disclosure of the probe sequence is highly desirable and strongly encouraged. However, since not all commercial pre-designed assay vendors provide this information, it cannot be an essential requirement. Use of such assays is advised against.
